# Development and Psychometric Properties of a health-promoting self-care behavior scale (HPSCB-S) in recovered patients from Drug Addiction

**DOI:** 10.1186/s12889-023-15311-9

**Published:** 2023-02-28

**Authors:** Mehrdad Assadian Narenji, Maryam Khazaee-Pool, Abedin Iranpour

**Affiliations:** 1 Department of Health Education and Promotion, School of Public Health, HIV/STI Surveillance Research Center, Institute for Futures Studies in Health, Kerman University of Medical Sciences, Kerman, Iran; 2 Department of Health Education and Promotion, School of Health, Health Sciences Research Center, Mazandaran University of Medical Sciences, Sari, Iran

**Keywords:** Design and psychometrics, Self-care, Recovered from addiction, West of Mazandaran

## Abstract

**Background and objectives:**

Drug addiction is a chronic and relapsing brain disease. Practicing self-care is one of the rules offered by therapists to improve the withdrawal process and prevent relapse. Based on the evidence, health beliefs, including Pender’s proposed model, are significantly effective in adopting self-care behaviors in patients. On the other hand, in order to evaluate preventive interventions regarding drug use and relapse of drug use; Having a good and appropriate tool is a special priority. Therefore, the present study aims to design and determine the psychometric characteristics of the questionnaire for measuring health-promoting self-care behaviors in patients recovered from drug addiction has been carried out in the west of Mazandaran province.

**Methods:**

The current study is a cross-sectional descriptive methodological research. In the first stage, the items and primary classes of the new tool were extracted based on the review of studies consistent with Pender’s self-care promotion and a questionnaire was designed. In the second stage, the psychometric characteristics of the designed questionnaire were examined using face validity, content validity, construct validity and reliability. In the construct validity, the number of participants was 245 for exploratory factor analysis and 203 for confirmatory factor analysis. In the reliability test, 25 people participated to check internal consistency and test-retest. Random sampling was done from 12 drug addiction treatment clinics in two cities of Tonkabon and Ramsar in the west of Mazandaran province during 2021–2022. Data were analyzed using SPSS and AMOS software version 23.

**Results:**

The exploratory factor analysis revealed 7 factors containing 29 item explained more than 61% of the total observed variance. The results of confirmatory factor analysis showed that the data fit the model. In the reliability test, Cronbach’s alpha coefficient indicated the appropriate internal consistency of the tool and retesting the tool with an interval of 2 weeks showed acceptable stability of the tool.

**Conclusion:**

The present study showed that the researcher-made questionnaire has good validity and reliability and can be used to measure self-care behaviors of patients who have recovered from addiction in order to provide appropriate solutions to prevent the relapse of drug use.

## Introduction

Drug addiction is a chronic and relapsing brain disease that is associated with seeking and continuing the compulsive use of drugs despite harmful and damaging consequences [1, 2]. Today, the problem of addiction has become a national problem, and nations and governments around the world are facing the problem of drugs and addiction to these drugs. According to United Nations statistics, the number of drug users in 2013 was between 155 and 250 million people, which is 3.5–7.5% of the population aged 15 to 64. In Iran, substance abuse and relapse is considered a health and social problem. Iran suffers from drug abuse due to its neighbors, especially Afghanistan, as the largest producer of drug in the world. A study by the United Nations Office on Drugs and Crime (UNODC) on opiate trafficking shows that Iran reported the largest opiate seizures worldwide in 2014 [3]. In 2018, Iran’s anti-narcotics headquarters estimated the number of drug addicts at around 2.8 million between the ages of 15–64[4].

Despite the progress achieved in addiction treatment, one of the major challenges in this regard is relapse [2]. Relapse means the inability and failure to continue a behavioral change and the initiation of the behavior following a period of withdrawal [5]. The risk of returning to drug use is always present, and this issue complicates the treatment of addiction [6, 7]. Many studies show the high prevalence of returning to addiction and the high rate of relapse in addicts after being released from prison or rehabilitation centers. Some studies show that at least 2 out of 3 patients who tried to quit opium relapsed after six months. Researchers have come to the conclusion that only 20–50% of patients can continue to stop using effectively after one year. In Iran, based on the available information, 50% of addicts who go to rehabilitation centers to quit addiction have had a history of at least one attempt to stop using, which shows that the rate of relapse after quitting drugs is very high [8].

Recovery is a time-consuming process that often requires changes in various areas of a person’s daily life, including physical, behavioral, intrapersonal and interpersonal, psychological and social areas [9]. Despite the development of different addiction treatments in Iran, a significant number of patients return to drug use behavior. Therefore, various relapse prevention interventions have been designed by therapists [10]. One of the rules offered by therapists to improve the withdrawal process and prevent relapse is the practice of self-care. Self-care means conscious, acquired and purposeful activities that people do in order to preserve life and maintain and improve their health. Self-care improves health and quality of life, increases patient satisfaction, increases the effectiveness of services, rationalizes the use of services, and reduces health cost. Despite its importance, self-care is one of the most neglected aspects of drug withdrawal recovery [11]. Considering the problems in improving self-care and health-promoting behaviors, it is necessary to use theories or models of behavior change in this field. One of the health models presented in this regard is the model proposed by Pender in 1982 [12]. Pender defines lifestyle as a pattern of daily life activities that seriously affects a person’s health and is influenced by demographic, environmental and social conditions Predictive and explanatory constructs of health behavior in Pender’s model include: perceived benefits of action, perceived barriers to action, perceived self-efficacy, feeling related to behavior, commitment to action, interpersonal influencers and situational influencers.

Based on the literature review, various studies have been conducted on addiction and its recurrence, inside and outside of the country. Salari and his colleagues in 2020, investigated the role of social support in drug-using patients in their psychometric study [13]. Habibi and her colleagues revised Bramson’s addiction self-efficacy questionnaire and determined its reliability again [14]. Iranpour and his colleagues psychometrically analyzed the risk factors and protective factors of drug use in Iran [15]. Khazaee-Pool and her colleagues conducted a psychometric study of the attitude questionnaire of users towards methadone maintenance treatment [16]. Moreover, in another study, Khazaee-Pool and colleagues investigated and psychometrically evaluated the Persian questionnaire on the time to relapse in users [10].

According to the investigations and review of many texts and sources in this field, so far, no tool has been designed to specifically measure health-enhancing self-care behaviors in patients who have recovered from drug addiction. Therefore, researchers are facing challenges in the process of designing educational interventions regarding the recent issue. Therefore, the need to use a tool for such a purpose is necessary.

On the other, Considering the high percentage of relapse among Iranian consumers, it is necessary to design a tool based on Pender’s model to assess the self-care status of people in the recovery phase. An assessment tool should be standardized, easy to use for the population it is designed for, and have validity and reliability [17].

Therefore, considering the influence of self-care behaviors on social, cultural and organizational conditions, as well as the lack of standard tools (native and appropriate to the contexts and characteristics of Iranian society), the present study was conducted with the aim of Designing and determining the psychometric properties of a standard tool for measuring health-enhancing self-care behaviors in patients recovered from drug addiction in the west of Mazandaran province of Iran during 2021–2022.

## Materials and methods

### Study setting and design

This research was carried out during a supplementary cross-sectional survey of the qualitative study, approved by the Ethics Committee of Kerman University of Medical Sciences [The code of ethics obtained is IR.KMU.REC.1400.054], and all patients completed informed written consent. Data collection started in September 2021 and ended in June 2022. The study was conducted in two phases. Firstly, item generation and scale development were performed by applying three approaches: a literature review, a qualitative method approach, and interviews with a panel of experts. In the second phase, the psychometric properties of the scale were evaluated by means of cross-sectional studies with users recovered from drugs. We performed exploratory factor analysis, confirmatory factor analysis, and assessed the internal consistency of the scale. Furthermore, test-retest reliability was evaluated among an independent sample of 25 drug users. Table 1 provides the descriptive characteristics of the participants from the two phases.

**Table 1:** Demographic characteristics of the study sample

Demographic characteristics	EFA sample (n = 245) Number (%)	CFA sample (n = 203) Number(%)
**Age**		
Less than 20 years	0 (0%)	0(%)
20–25 years	8(3.3)	9(4.4)
26–30 years	19(7.8)	13(6.4)
31–35 years	23(9.4)	21(10.3)
More than 25 years	191(78)	160(78.8)
Mean (SD)	46.94(13.14)	45.44(13.26)
Range	21–83	20–85
**Gender**		
Woman	21(8.6)	16(7.9)
Man	221(90.2)	187(92.1
**Educational level**		
Illiterate	10(4.1)	8(3.9)
Primary	59(24.1)	48(23.6)
Middle	86(35.1)	74(36.5)
High	60(24.5)	53(26.1)
Academic	26(10.6)	19(9.4)
**Employment status**		
Full time employee	53(21.6)	43(21.2)
Part-time	16(47.3)	77(37.9)
Retired	30(12.2)	25(12.3)
Unemployed	30(12.2)	42(20.7)
Homemaker	15(6.1)	15(7.4)
Collegian	0(0)	1(0.5)
**Marital status**		
Married	187(76.3)	144(70.9)
Divorced	16(6.5)	9(4.4)
Widow	2(0.8)	6(3)
Single	39(15.9)	44(21.7)
**Place of life**		
City	158(64.5)	145(71.4)
Village	74(30.2)	58(28.6)
**Income (in Iranian Rial)**		
10–50 million Rial	191(78)	110(54.2)
50–100 million Rial	32(13.1)	43(21.2)
100–150 million Rial	0(0)	10(4.9)
Above 150 million Rial	1(0.4)	2(1)
No income	21(8.6)	37(18.2)

### Sample size and participants

This study was conducted on patients recovered from addiction who referred to 12 addiction treatment centers in two cities of Tankabon and Ramsar from the west of Mazandaran province. Sampling was done randomly. At first, the total number of clinics in two cities was counted. Then, 6 clinics were randomly selected for exploratory factor analysis. In the confirmatory factor analysis, these 6 clinics were excluded and 6 other clinics were considered. In total, 448 people completed the questionnaire. Inclusion criteria include for all sample were: consent to cooperate and not having acute psychiatric diseases such as psychosis.

### Psychometric testing

#### Phase 1: item generation

In this phase, by systematically reviewing the texts, using databases, all the articles that existed in the period of 2010 to 2021 regarding tools in the field of addiction, relapse, self-care and the Pender model in addiction were searched and studied. Then, the primary items were extracted based on Pender’s model structures and evaluated by the research team to ensure accuracy and to find overlapping and repetitive items. Duplicate items were removed. The number of items was initially reduced to 60 and at the end of the first phase of the study to 50 items.

#### Phase 2: validity and reliability evaluation of the designed tool

To examine the psychometric properties of the tool, it was assessed its content, face, and construct validity as well as its reliability and stability.

### Content validity

A 50-item questionnaire was used to determine content validity. Both qualitative and quantitative approaches were used to assess the content validity. In the qualitative phase, a panel of experts (n = 10), including health promotion experts, epidemiologists, psychologists specializing in tool design and specialists in drug-use control, measured the content validity of the tool, evaluating its phrasing, grammar, wording, item allocation, and scaling. In this section, the opinions of the panel members were collectively written for each item in the considerations section and sent to the professors, and after consultation, a review was made and the text of some items was changed and corrections were made in the tool. The next version of the items was prepared. Then the panel members studied the questionnaire quantitatively. In the quantitative phase, the content validity index (CVI) and content validity ratio (CVR) of the tool were assessed for each item and again sent to professors for decision. The CVI was assessed by asking the experts to rate each item according to its simplicity, relevance, and clarity [18] on a scale from 1 = not relevant, simple, or clear to 4 = very relevant, simple, and clear. The CVI was measured as the proportion of items on the questionnaire that achieved a rating of 3 or 4 [19, 20]. A CVI score of 0.79 or above for each item was considered to be acceptable. Items with a CVI score between 0.70 and 0.79 were revised and items with a CVI score less than 0.70 were excluded. The essentiality of each item in the questionnaire was evaluated by the CVR. To measure the CVR, the specialists rated each item as 1 = essential, 2 = useful but not essential, or 3 = not essential. Then, according to the Lawshe table, items with a CVR score of 0.62 or greater were determined to be acceptable and were maintained (20, 21, 22).

### Face validity

Both a qualitative and a quantitative approach were used to measure face validity. First, the items were arranged based on the results of content validity, then questionnaire was prepared in two separate forms: A- to determine the qualitative face validity (examining the level of difficulty, appropriateness, ambiguity and inadequacy) and B- to determine the quantitative face validity (5-option Likert scale to check the importance of the items) and given to 10 members of the target group who were randomly selected from addiction treatment clinics. Afterward, the impact score (importance × frequency) was measured to determine the percentage of addicted persons who recognized an item as important or quite important on a 5-point Likert instrument. An item was determined to be suitable if it had an impact score of 1.5 or higher (21, 22, 23).

### Construct validity

#### Exploratory factor analysis

An EFA was performed to find the main factors of the tool. The sample size was assessed a priori and was estimated according to the number of items in the instrument multiplied by 7 (7 × 35 = 245) [24]. The participants were recruited from the 6 clinics. According to the type of study, which was self-care, questionnaires were prepared with a 5-point Likert scale (never, rarely, sometimes, usually, and always) and distributed among 245 people. After completing the questionnaires and entering the data into SPSS software, a principal component analysis with varimax rotation was applied to extract the main factors. Factor loadings of ≥ 0.40 were considered acceptable. Two primary tests were used to check the fit of the data for exploratory factor analysis: The Kaiser-Meyer-Olkin (KMO) test to assess the adequacy of the sample and Bartlett’s test of sphericity to assess the justification of factor analysis [24].

SPSS software version 23 was used to Exploratory factor analysis.

#### Confirmatory factor analysis

A CFA was performed to measure the coherence between the model and the data. First, the items of the questionnaire were rearranged based on the results of exploratory factor analysis in 5 Likert scales: never, rarely, sometimes, usually and always. Sampling was done again. Assigning 7 individuals to each item, a sample size of 203 was estimated. After collecting the questionnaires, the model was estimated by entering the data in software. Then, with the help of several fit indices, which included: the relative chi square (CMIN/df); comparative fit index (CFI), normed fit index (NFI), Tucker Lewis index (TLI), Incremental Fit Index (IFI), Relative Fit Index (RFI) and root mean square error of approximation (RMSEA), the model was evaluated.

Usually, as the first index, the chi-square value is reported, but it should be noted that this index alone does not indicate whether the model is acceptable or unacceptable. The significance of this index can be due to the sensitivity to the large sample size. Mostly, in such cases, the relative chi-square (CMIN/DF) is the basis of judgment. Values from 1 to 5 are acceptable for this index, and values between 2 and 3 are considered very good [24].

CFI, NNFI, and NFI values range from 0 to 1, but values equal to or greater than 0.80 are generally considered satisfactory model fits [21]. Of course, in many sources, a value higher than 0.9 is considered as a desirable and very good value. The RMSEA index varies between two values, zero and one, and is known as one of the bad fit indices. Therefore, the lower its value and closer to zero, the more acceptable it is. A numerical value below 0.05 indicates a good model fit, but values between 0.06 and 0.08 also indicate an adequate and good fit. The value of RMSEA between 0.08 and 0.10 indicates the average fit of the model (10, 21). AMOS software version 23 was used to Confirmatory factor analysis.

### Reliability

in this research, the reliability of the tool was investigated by studying the internal consistency (via calculating Cronbach’s alpha coefficient) as well as assessing the stability (by test-retest method).

1- Cronbach’s alpha represents the appropriateness of a group of items that measure a construct. Cronbach’s alpha coefficient was calculated for each factor as well as the entire questionnaire. Conventionally, the existence of minimum Cronbach’s alpha equal to 0.6 is desirable for descriptive studies, but usually the appropriate internal validity index is considered between 0.7 and 0.8, which shows that the questionnaire has acceptable reliability (10, 24, 25). In this study, Cronbach’s alpha above 0.7 was considered acceptable. In order to measure this coefficient, the modified questionnaire was distributed among 25 people of the target group.

2- Stability is considered an important part of tool reliability and represents the degree of similarity of results in repeated measurements. To evaluate the stability of the questionnaire, the test-retest method was used. In this method, 25 people were randomly selected from the target group and completed the questionnaire for the second time with an interval of two weeks. The most acceptable test to determine stability is the Intraclass Correlation Coefficient (ICC) test. For this purpose, the data obtained from the test-retest method was entered into software and then ICC was calculated. ICC values equal to or greater than 0.40 are considered satisfactory (r values between 0.81 and 1.0 are excellent, those between 0.61 and 0.80 are very good, those between 0.41 and 0.60 are good, those between 0.21 and 0.40 are fair, and those between 0.0 and 0.20 are poor) (10, 26). ICC was calculated for each factor separately and also for the whole questionnaire. SPSS software version 23 was used to calculate Cronbach’s alpha coefficient and ICC.

## Results

### Demographic characteristics of the participants

448 participants completed the questionnaire, who were in the age range of 20–85 years. The average age of the patients was 46.25 with a standard deviation of 13.20 years and the age range was 85 − 20 years. 351 people (79.81%) of the participants belonged to the age category above 35 years. In the age group under 20 years, the number of participants was zero. 37 (8.3%) of the participants were female and 408 (91.7%) were male. In terms of job status and employment, the largest number (193 people (43.2%)) were part-time workers. The number of participating students was 1 (0.2%). In terms of education level, the highest percentage was in the middle school/cycle level (160 people (36.1%)). In terms of marital status, the largest number belonged to the married group (331 people (74%)). Also in terms of place of residence 303 people (69.7%) lived in the city and 132 people (30.3%) lived in the village. The monthly income of most people (301 people (67.2%)) was in the range of 10–50 million Rials (Table 1).

The age of the first attempt to use drugs in 168 people (38.1%) was in the age range of 20–29 years, and the rest were in other age groups. Most of the people (260 people (58.2%)) used opium, burnt and juice as a drug before quitting. Before recovery, most people (427 people (96.4%) used drugs non-injectively. The number of times of consumption before cleansing was 2–3 times a day in 301 people (69.4%). 192 people (43.2%) reported a history of drug use in family members. Also, 329 people (73.6%) reported a history of drug use in close and intimate friends. A number of 320 people (71.4%) continue to smoke after being clean. But the consumption of hookah, oral tobacco and alcoholic beverages showed a lower percentage (143 people: 31.9% hookah consumption; 25 people: 5.6% oral tobacco use such as Nas and 115 people: 25.7% reported alcohol consumption did). In a high percentage of people (323 people (72.1%)), the number of months clean from substances was more than 9 months. The highest number of attempts to quit was once (203 people (46.8%). In terms of physical and mental health status, a lower percentage was referring to a doctor (126 people (28.1%)) and a psychiatrist (71 people (8%). /15)) were reported respectively.

### Content validity

The Average CVI and CVR index, two measures of content validity, were good (CVI = 0.91 and CVR = 0.64). According to the results, and in consultation with the professors, the number of items was reduced from 50 items to 35 items, and 15 items were excluded from the study due to being unrelated or repetitive.

### Face validity

In both qualitative and quantitative models, the impact factor for all items was higher than 1.5, and therefore all 35 items were considered suitable for further analysis.

### Construct validity

After the item analysis, the 35 remaining items were considered to estimate the construct validity using exploratory factor analysis (EFA) and confirmatory factor analysis (CFA).

### Exploratory factor analysis

The measured KMO was 0.805, and the Bartlett’s test of sphericity was significant (χ2 = 3061.999, p < .001), so the sufficiency of the data to perform the factor analysis was confirmed and there was a significant correlation between the variables, therefore the data had the appropriateness for this analysis.

**Fig. 1:**
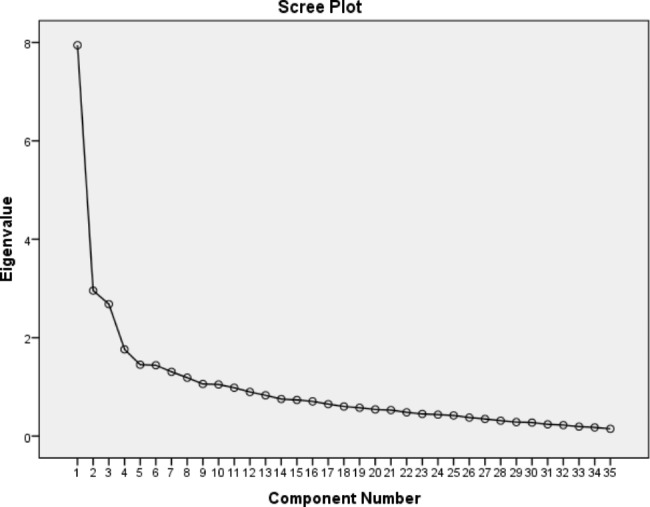
Slope diagram of the range of factors (scree plot) in factor analysis

In order to extract the factors, the principal component analysis method was used. The results of the first stage analysis showed that the eigenvalue in the Extraction column of item number 26 is equal to 0.358, which means that 35% of the variance of item number 26 scores is the variance of the common factor and is less than 0.4. Therefore, item number 26 (my family insists on the treatment process until my complete recovery) was removed from the analysis process in the second stage of the analysis. Considering the results of eigenvalue, the second stage of analysis continued by removing the mentioned item. In second stage of exploratory analysis, 10 factors were extracted, had eigenvalues greater than 1 and in total, explained 65.24% of the total variance. Additionally, the scree plot showed a 10-factor solution (Fig. 1). In this step, two of the factors had only two items. Therefore, the third stage analysis with Varimax rotation was performed. The results of this step of analysis showed that 7 factors had eigenvalues greater than 1 and in total, 61.97% of the total variance was explained by these 7 factors. Content of factors and items in this analysis shows in Table 2. On the other words, this factor solution was explored by repeatedly assessing the item performance with elimination of the items in a step-by-step process. After eliminating the items with factor loadings below 0.4, a final factor solution that consisted of 29 item loading on 7 distinct factor was extracted and 6 items numbered 1, 6, 16, 19, 26, 30, deleted from the questionnaire.

**Table 2:** Loading the eigenvalues of items after Varimax rotation in Exploratory factor analysis (n = 245)

Items	Factor 1	Factor 2	Factor 3	Factor 4	Factor 5	Factor 6	Factor 7
Q11. Keeping clean from drug use is the first priority in my life.	**0.734**	0.144	− 0.017	0.000	− 0.124	0.167	− 0.024
Q12. I feel responsible for my life, my family, those around me and my friends.	**0.710**	0.006	− 0.002	0.102	− 0.001	0.107	0.019
Q18. Seeing people who have been cleansed, I become more determined to continue cleansing.	**0.696**	0.238	− 0.011	0.337	0.050	0.010	− 0.067
Q10. I try to improve my relationships with healthy friends and relatives.	**0.578**	0.263	0.182	0.001	− 0.022	0.304	0.232
Q9. I avoid situations and situations that remind me of drug use or the temptation to use it.	**0.541**	0.027	0.280	0.142	0.141	0.295	0.203
Q17. With my strong will, I can accept my past bad days.	**0.500**	0.087	0.134	0.434	0.043	0.035	0.349
Q13. By strengthening self-confidence, I try not to pay attention to negative words, stigma, and….	**0.453**	0.121	0.196	0.213	− 0.096	− 0.132	0.408
Q35. If I use drugs again, I may be kicked out by my family.	0.076	**0.851**	0.026	0.026	0.092	0.101	0.014
Q34. If I use drugs again, I may no longer be trusted by anyone.	0.135	**0.833**	0.076	0.106	0.096	0.031	0.112
Q33 If I use drugs again, I may lose my job/or not be able to find a suitable job.	0.093	**0.806**	− 0.035	− 0.014	0.090	0.018	0.004
Q32. If I use drugs again, my relationship with my family and close people will be at risk.	0.145	**0.789**	0.021	0.116	0.109	0.107	0.032
Q8. In my spare time, I try to keep myself busy with suitable activities such as sports.	0.138	− 0.015	**0.858**	0.176	0.039	− 0.001	0.103
Q7. When I feel anxious, I try to occupy myself with positive and enjoyable activities.	0.077	− 0.043	**0.808**	0.209	0.171	0.142	0.108
Q5. I have a regular daily schedule to continue my recovery.	0.117	0.092	**0.684**	0.037	0.110	0.420	0.110
Q22. During the period of purity and recovery, I do my best to solve problems.	0.216	0.006	0.115	**0.687**	0.035	0.186	0.138
Q25. Whenever I need something, I can have the support of my family.	0.236	0.072	− 0.018	**0.608**	0.290	0.019	0.123
Q24. Whenever I encounter a problem, I can substitute several suitable solutions.	0.073	− 0.063	0.316	**0.601**	0.284	0.004	0.343
Q20. If I have a feeling of need and physical pain, I replace it with positive activity.	0.018	0.226	0.442	**0.593**	− 0.194	0.109	0.061
Q21. When I have physical pain or discomfort, I tell my doctor or counselor.	0.093	0.107	0.165	**0.560**	0.199	0.359	− 0.128
Q27. My family and people around me know what measures are necessary to stay clean.	0.211	0.126	− 0.026	0.028	**0.749**	0.054	0.137
Q28. I have at least one trusted friend whom I can support in any situation.	− 0.021	0.153	0.025	0.161	**0.724**	0.189	− 0.040
Q29. My friends are aware of my treatment process.	− 0.231	0.022	0.165	0.127	**0.709**	− 0.062	0.089
Q31. There are enough recreational and entertainment facilities in my living environment.	− 0.104	0.202	0.357	0.077	**0.466**	0.046	0.122
Q2. I consult a counselor or a guide if I have a craving to use drugs.	0.055	0.071	0.057	0.195	0.113	**0.694**	0.045
Q4. If I have an illness, I use my medical services and other medicines on time.	0.235	0.064	0.188	0.081	0.089	**0.675**	− 0.049
Q3. By focusing on my work and life, I prevent myself from slipping again.	0.206	0.121	0.073	0.071	− 0.161	**0.591**	0.463
Q15. I have the skills to overcome drug addiction.	0.072	0.025	0.086	0.087	0.255	0.097	**0.777**
Q23. By strengthening my will, I can handle unexpected and tempting situations.	− 0.053	0.155	0.128	0.366	0.173	0.359	**0.546**
Q14. I feel that I have the necessary preparation for the period after the treatment.	0.442	− 0.004	0.236	0.070	− 0.023	− 0.145	**0.458**

Thus, Factor 1 included items 9, 10, 11, 12, 13, 17, 18; Factor 2 included items 32, 33, 34, 35; Factor 3 included items 5, 7, 8; Factor 4 included items 20, 21, 22, 24, 25; Factor 5 included items 27, 28, 29, 31; Factor 6 included items 2, 3, 4 and Factor 7 included items 14, 15, 23. After reviewing and exchanging opinions, the following names were chosen for the factors in according to the factor loading of the first item in each dimension and its correspondence with self-care patterns and Pender’s model: first factor: self-efficacy, second factor: perceived sensitivity, third factor: lifestyle modification, fourth factor: responsibility, fifth factor: constructive support, sixth factor: Health literacy and the seventh factor: self-control.

### Confirmatory factor analysis

**Fig. 2:**
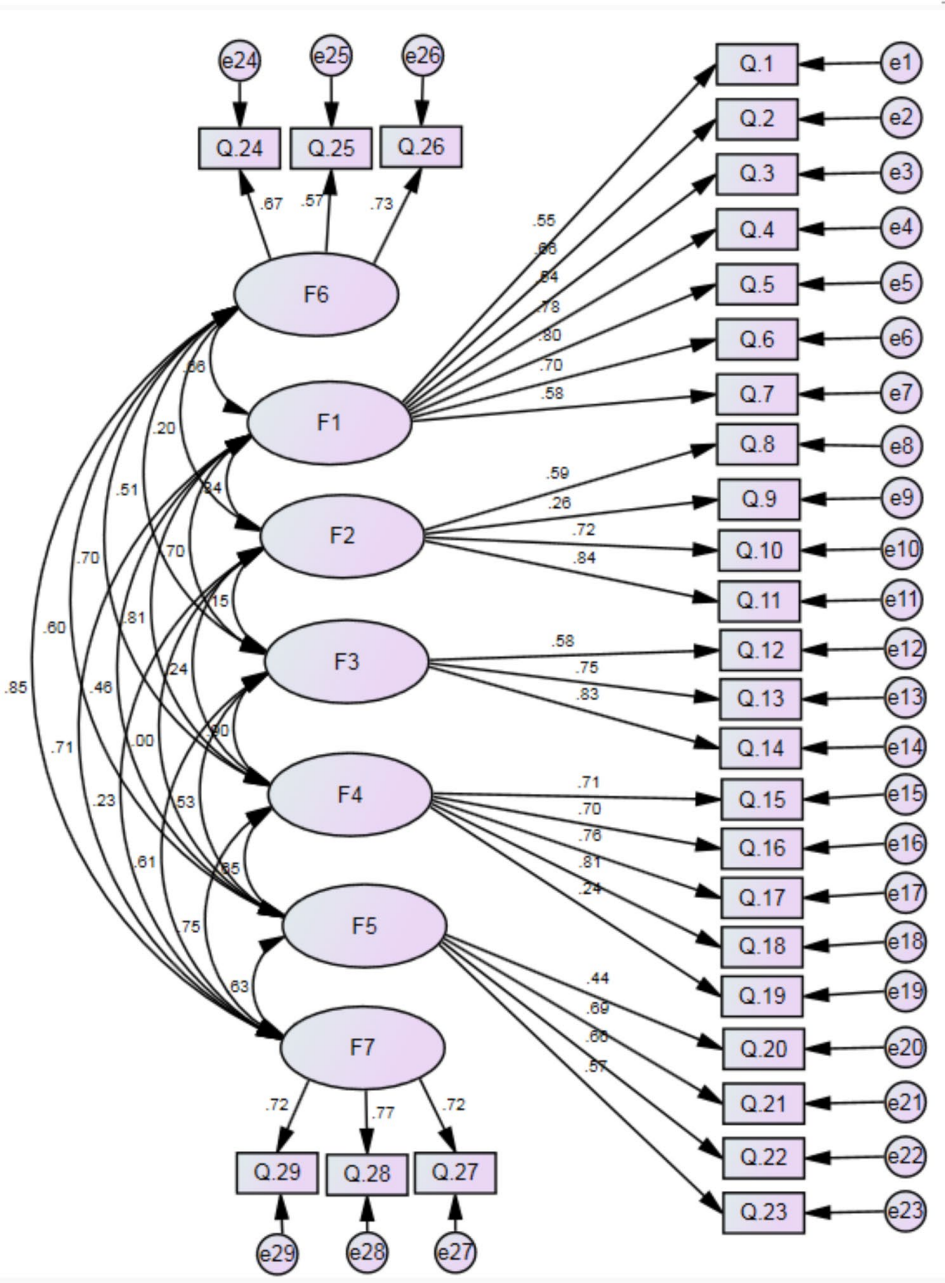
- Confirmatory factor analysis diagram of the tool using Amos software

At this stage, using the results of exploratory factor analysis, a suitable model was formulated and factor loadings in the model were examined and the model was measured and estimated through the software. Figure 2 shows the best model fit for the questionnaire. Covariance matrices were used, and fit indices were calculated. Most of the fit indices were acceptable. The relative chi-square (χ2/df) was equal to 1.911 (p < .001). The RMSEA of the model was 0.061. Indices of the model, including CFI, TLI and IFI, were more than 0.8 (0.86, 0.83 and 0.86 respectively). Indices RFI and NFI, were more than 0.7 (0.70 and 0.75 respectively). In total. the results showed that the developed model is appropriate and has a good fit, and this tool is sufficient to be used in future research.

### Reliability

The internal reliability was measured using Cronbach^,^s alpha. All values were above acceptable thresholds, with alpha = 0.95 for the total questionnaire and alpha^,^s ranging between 0.83 and 0.96 for factors. Also, ICC values was 0.76 for the total questionnaire, with ICC values ranging from 0.66 to 0.78 for the subscales and factors. The finding is displayed in Table 3.

**Table 3:** Reliability estimates for scale and subscales of questionnaire

Factor	Name of Factor	Number of Items	Cronbach’s α(n = 25)	ICC (n = 25)
1	self-efficacy	7(9, 10, 11, 12, 13, 17, 18)	0.96	0.70
2	perceived sensitivity	4(32, 33, 34, 35)	0.88	0.73
3	lifestyle modification	3(5, 7, 8)	0.87	0.78
4	responsibility	5(20, 21, 22, 24, 25)	0.86	0.68
5	constructive support	4(27, 28, 29, 31)	0.83	0.66
6	Health literacy	3(2, 3, 4)	0.83	0.70
7	self-control	3(14, 15, 23)	0.90	0.71
**total**		**29**	**0.95**	**0.76**

## Discussion

The present study was conducted with the aim of designing and psychometrically measuring self-care behaviors that promote health in patients recovered from drug addiction in the west of Mazandaran province during 2021–2022. Overall, the findings showed that the psychometric properties of the designed tool are good and acceptable. This research is considered an innovation due to the design and psychometrics of the self-care questionnaire in patients recovered from addiction. Existing research in Iran and other countries focused on other dimensions of addiction or relapse or used pre-designed questionnaires in the studies. For this reason, this tool cannot be easily compared with other tools. According to the objectives, the obtained results can be discussed and concluded in two demographic and psychometric sections.

Overall, the study found satisfactory psychometric properties for the tool, with the CVI and CVR indicating that its content validity was good. Furthermore, the results of the EFA and CFA revealed a good structure, with the EFA showing that the seven-factor structure of the tool (HPSCB-S) accounted for 61.971% of the total observed variance. In this way, the tool created by the researcher that named HPSCB-S includes 29 items (questions) and 7 dimensions: 1- self-efficacy (7 items), 2- Perceived sensitivity (5 items), 3- lifestyle modification (3 items), 4- Responsibility (5 items), 5- Constructive support (4 items), 6- Health literacy (3 items) and 7- Self-control (3 items), were confirmed. The CFA also examined whether coherence exists between the information and the theoretical structure. The CFA revealed good fit indices for the existing model and demonstrated the acceptable convergent validity of the tool. Additionally, acceptable levels of the Cronbach’s alpha and the ICC were found, and the good stability and reliability of the tool were demonstrated. The results of the ICC showed that the most reliable factor is the lifestyle modification factor and then the perceived sensitivity factor, which showed the lowest measurement standard error and the highest value of the ICC. Our results clearly show that the tool has appropriate stability in the short term; however, it has yet to be determined whether it is stable over the long term. Overall, the findings indicate satisfactory psychometric properties for the tool.

According to the 7 dimensions obtained in this study, it can be said that, like other studies, improvement in self-care behaviors occurs through several factors and thus shows the role of individual differences, which emphasizes on conducting more studies in this population. In addition to this, the development of theory-based questionnaires and scales can be an important precondition for the evaluation of any educational program. In the discussion of the results obtained in the construct validity section and the named dimensions, it can be said that there is no specific agreement about the root causes of substance abuse, and the etiology of this phenomenon is very important for designing effective programs to prevent substance abuse.

The adoption of self-care programs in the field of addiction and relapse is always a challenging issue for recovering patients, physicians and health care providers in this field [27]. Self-care means conscious, acquired and purposeful activities that people do in order to preserve life and improve their health [28]. Pender’s model is one of the comprehensive models that can be used to plan for health promotion in the field of addiction [29]. Despite the importance of this model, its constructs have not been sufficiently considered as a causal model for self-care health behaviors in populations of patients recovering from addiction. Therefore, it seems necessary to design a reliable and valid questionnaire in this field.

Several studies have investigated the effect of self-care program based on the Pender model on the quality of life of patients. But as it was said before, there are not many resources available in the field of questionnaire design in addiction treatment based on Pender’s model. In a study conducted by Bradbury-Golas in 2013, A comparative, descriptive, no experimental study of a convenience sample of 74 subjects explored health promotion practices of recovering opiate-dependent drug users and compared them with those of abusing opiate-dependent drug users using the Pender’s Health-Promoting Lifestyle Profile II. Findings showed that recovering opiate-dependent drug users practiced more health promoting behaviors in all health promotion areas with significant differences. Results showed that Nutrition, physical activity, stress management, spiritual growth, interpersonal relationships, and avoidance of high-risk situations are essential health-promoting and relapse prevention behaviors that recovering individuals need to maintain a healthy lifestyle [30]. Ghasem and et al., in 2014, investigated the effects of educational intervention on health-promoting lifestyle and health-related life quality of methamphetamine Users and Their Families based on Pender model. In this study, a demographic checklist and a standard questionnaire covering health-promoting lifestyle, health-related QoL, self-efficacy, perceived affect, perceived social support, and perceived barriers dimensions were used to gather required data. Analysis of results showed that after adjusting for effects of pretest scores, the difference between mean post-test scores of all constructs of Pender’s health promotion model were significant [31]. According to the mentioned points, below we try to compare as much as possible the closeness of the obtained dimensions with Pender’s model and other close studies.

The first named dimension is self-efficacy, which is consistent with the perceived self-efficacy structure of the Pender model. Self-efficacy is one of the variables that is related to substance abuse. This word is one of the most effective topics in behavior change theories, especially Bandura’s social cognition theory, and its effects have been reported many times in various fields, including substance abuse. ‘I can’, form the basis of the concept of self-efficacy. Therefore, increasing self-efficacy can protect a person from deep pressures [32]. Belief in self-efficacy is one of the psychological factors that is effective in the success of treatment. Self-efficacy belief is a cognitive-motivational force that determines the appropriate coping level of people when their skills and abilities are under pressure. Weak self-efficacy beliefs destroy people’s ability to solve problems. Several studies claim that self-efficacy plays a special role in predicting treatment outcomes and improving the health of substance-dependent individuals. For example, in study of Ibrahim and et al. in 2011, a significant negative relationship was observed between self-efficacy and addiction relapse. People with low self-efficacy are more likely to continue using drugs (33, 34). Torrecillas and et al. in 2015 showed that self-efficacy is indirectly related to the amount of drug dependence [35].

The next dimension is perceived sensitivity. This dimension can be consistent with the structure of perceived barriers of Pender’s model. In a study conducted by Tavakli Gouchani and et al. in 2014, after reviewing the available articles and documents, they identified 27 factors and motivations related to the action and continuation of drug withdrawal, which include a negative attitude towards continued use., external pressures, Relationships between children and parents, family insistence, consequences of consumption, fear of legal trouble, disgrace and loss of job, maintenance of family connections, and presence of family support. Then these cases were classified in different structures. One of the constructs named in this research was perceived sensitivity, which was defined as a person’s understanding of the degree of vulnerability to a disease, and based on this definition, some of the aforementioned factors for quitting addiction, such as awareness of its negative effects on oneself and others, fear of legal troubles, fear of dishonor, fear of job loss, creating family problems, creating employment problems, etc. were placed in this structure [36].

The third dimension of the designed tool was named lifestyle modification. This dimension can be consistent with the structure perceived benefits of action of Pender’s model. In a review article in 2018, Thompson and et al., discussed the role of physical activity in the prevention and reduction of alcohol and drug addiction treatment and showed that physical activity as an alternative, low-cost and accessible method can reduce the amount of substance use. In this study, the effect of physical activity and its potential on reducing the risk of relapse and reducing consumption was investigated and useful results were obtained [37]. The results of Jafari and Gurbanalipour’s study in 2015 also showed that teaching coping skills and lifestyle significantly prevents relapse in people under training [38]. In the research of Rajabi and Moghaddis Tabrizi, the effect of exercise training in preventing relapse of morphine use in patients under methadone treatment was investigated. The results showed that aerobic exercises were effective in reducing the recurrence and increasing the duration of the treatment and presented it as a suitable treatment solution [39]. In 2020, Cabrera showed in his article that mental and physical health improved by doing physical exercise as part of a healthier lifestyle, and patients could avoid the immediate reinforcing symptoms of drug use and reduce cravings and causes a decrease in the risk of relapse [40].

Another introduced dimension is responsibility. This dimension can be consistent with the structure commitment to action of Pender’s model. In a study done by Akbari in 2019, The effectiveness of cognitive behavioral therapy group on patients referred to addiction treatment centers was investigated. Cognitive behavioral therapy can be effective on the restraint of patients who are quitting drugs and prevent relapse. One of the components of restraint is responsibility. The results showed that this treatment can increase people’s responsibility compared to before the treatment [41]. In other words, the inability to accept responsibilities causes these people to be unsuccessful in addiction treatment. Karimi and Kiani’s study in 2022 showed that the three characteristics of clients, including assertiveness, responsibility, and life orientation, are important variables related to success in the durability of addiction treatment [42].

The fifth named factor is constructive support (family and friends), which can be placed in the structure of interpersonal influencers of Pender’s model. In the discussion of constructive support, the term social support can be used. It has been determined that the dimensions of social support are the strongest coping force for facing stressful situations. Social support is defined as a person realizing that others care about their feelings and are considered as a valuable person, which can be evaluated in two ways: received social support and perceived social support. Many studies have shown that perceived social support plays a role in the prevention, treatment and prevention of relapse. Davis and Jason concluded that substance abstinence was positively related to receiving social support [43]. Social support can reduce stress and increase self-efficacy and self-esteem. In 2020, Salari et al., in a study, examined the validity of the social support measurement tool in Iranian drug users, and in this study, while confirming the mentioned tool, they considered social support as a key factor in the treatment and rehabilitation process of people who They use drug [13]. In 2019, Khazaee-Pool et al., in their study on understanding the process of relapse in female drug users, concluded that, along with many factors, lack of family support and social conditions and rejection by Mitwand’s friends are important reasons for relapse [4]. In 2017, Nikmanesh and colleagues discussed the importance of the two items of self-efficacy and social support in these patients in their article entitled the role of self-efficacy beliefs and social support in predicting relapse [34].

Health literacy (sub-dimension of decision-making) is another dimension achieved. This dimension can be consistent with the structure perceived benefits of action of Pender’s model. In the study of zareban and et al., in 2015 on the psychometrics of the adult health literacy questionnaire, the dimensions of this questionnaire and its questions were evaluated. The last dimension of this questionnaire was the decision dimension [44]. The questions that were placed in our research questionnaire in the dimension of health literacy were very similar to the questions in the decision-making dimension of the said questionnaire, which can be the reason for choosing the name of health literacy for dimension 6 of our study. Health literacy is cognitive, social skills and the ability of people to understand and use available information to promote and maintain good health [44]. In 1400, mosalman et al., studied the prediction of readiness for addiction based on health literacy among college students. The results of this study showed that readiness for addiction had a significant negative relationship with the score of health literacy and its components (which included access, skill, reading, understanding, evaluation, decision-making and use of information) [45]. Deegan and colleagues in 2019 showed that low to moderate levels of health literacy were common among those in substance abuse treatment [46]. Also, the results of Karimi et al.‘s research in 2017 indicated that women with higher health literacy have a lower tendency to addiction [47].

The next named dimensions are self-control. This dimension can be consistent with the structure feeling related to behavior of Pender’s model. In the explanation of this aspect, it can be said that an important factor in the failure of drug addicts to quit is the craving for drugs. In fact, the desire to consume is one of the factors that perpetuate addictive behaviors and plays an important role in the phenomenon of return or relapse. Craving is an uncontrollable desire that, if not satisfied, can lead to a lot of psychological and physical suffering in drug addicts. Therefore, it seems that craving is controlled by cognitive-emotional processes. Self-control is one of the most important factors that protect a person against cravings. Self-control means how stable or flexible a person acts in the current situation. If the source of control is internal, these people will leave faster and the probability of returning is less [48]. HajiHasani et al.‘s research showed that people with more self-control are less prone to addiction [49]. Also, in the study of Yang et al., in 2019 in China, it was found that self-control increases the self-efficacy of drug withdrawal in patients [50].

## Conclusion

Generally, the results of this study will be useful for people who are part of a drug control program and participate in it. The present designed questionnaire has the necessary validity and reliability and it is hoped that it will be used in the investigation of self-care behaviors of patients recovered from addiction in other regions of the country. It is suggested that this questionnaire be used on a wide level so that, while measuring the self-care of improved patients, the efficiency of the questionnaire is also reevaluated.

Among the limitations of the study, the following can be mentioned:1- Sampling from limited areas and a province and completing questionnaires in the form of self-report 2- Non-cooperation of a large number of people due to cultural and social conditions and fear of stigmatization and loss of social image 3- The possibility of providing incorrect answers by people or providing a false image by people of themselves in this type of questionnaire.

## Data Availability

The datasets produced and analyzed throughout the current study are not publicly available because of the need to maintain the participants’ privacy. However, on reasonable request, they may be available from the corresponding author.

## Abbreviations

UNODC: United Nations Office on Drugs and Crime
CVI: Content Validity Index
CVR: Content Validity Ratio
EFA: Exploratory factor analysis
CFA: Confirmatory factor analysis
CFI: Comparative Fit Index
RMSEA: Root Mean Square Error of Approximation
NFI: Normed Fit Index
NNFI: Non-Normed Fit Index
ICC: Intraclass Correlation Coefficient
KMO: Kaiser-Meyer-Elkin
